# Explaining interpersonal differences in COVID-19 disease prevention behavior based on the health belief model and collective resilience theory: a cross-sectional study from Bolivia

**DOI:** 10.1186/s12889-022-13068-1

**Published:** 2022-05-31

**Authors:** Boris Christian Herbas-Torrico, Björn Frank

**Affiliations:** 1 Exact Sciences and Engineering Research Center (CICEI), Bolivian Catholic University San Pablo, M. Marquez Street and Jorge Trigo Andia Park - Tupuraya, Cochabamba, Bolivia; 2 Faculty of Commerce, Waseda University, 1-6-1 Nishi-Waseda, Shinjuku-ku, Tokyo, 169-8050 Japan

**Keywords:** Coronavirus, COVID-19, Disease prevention, Hygiene, Social distancing, Surgical mask

## Abstract

**Background:**

Governments have attempted to combat the COVID-19 pandemic by issuing guidelines for disease prevention behavior (e.g., wearing masks, social distancing, etc.) and by enforcing these guidelines. However, while some citizens have complied with these guidelines, others have ignored them or have even participated in large-scale protests. This research aims both to understand the causes of such variation in citizens’ adherence to government guidelines on disease prevention behavior and to extend the scientific literature on disease prevention to account for the collective resilience of a society to diseases. Thus, this research draws on the health belief model and collective resilience theory to develop hypotheses about the determinants of a citizen’s disease prevention behavior. These hypotheses deal with how citizens’ vulnerability, attitudes toward disease prevention, and social orientation are associated with COVID-19 prevention behaviors.

**Methods:**

From March 24 to April 4, 2020, a cross-sectional online survey was conducted in Bolivia. It included questions on demographic characteristics, chronic health problems, emotional burden, attitudes towards preventive behaviors, trust in public institutions, and culture. Among 5265 participants who clicked on the survey, 1857 at least partially filled it out. After removing data with missing responses to any variable, the final sample consists of 1231 respondents. The collected data were analyzed using hierarchical linear modeling.

**Results:**

Regarding a citizen’s vulnerability, chronic health problems have a U-shaped association with disease prevention behavior. Moreover, age, female gender, and worries have positive associations with disease prevention behavior, whereas depression showed a negative association. Regarding attitudes toward disease prevention, trust in public institutions, and attitudes toward social distancing, a government-imposed lockdown and the enforcement of this lockdown showed positive associations with disease prevention behavior. Regarding social orientation, individualism and collectivism both have positive relationships with disease prevention behavior.

**Conclusions:**

In the COVID-19 pandemic, a citizen’s low vulnerability, weak social orientation, and beliefs about low benefits of disease prevention behavior are associated with poor compliance with guidelines on disease prevention behavior. More research on these associations would help generalize these findings to other populations and other public health crises.

**Supplementary Information:**

The online version contains supplementary material available at 10.1186/s12889-022-13068-1.

## Introduction

In late 2019, the world discovered a new virus called SARS-CoV-2, which can cause a disease called COVID-19 [1]. As one of the most significant worldwide health crises of the century, the COVID-19 pandemic has posed challenges in practically every aspect of human functioning, including the global economy and the entire social system [2, 3]. As of February 21, 2022, this pandemic had infected over 424 million people [4], with over 5.9 million people dying from the infectious disease [5], making it one of the deadliest pandemics of the century [2, 6–8]. To contain its spread, governments worldwide have issued guidelines for disease prevention behaviors, such as face mask use, frequent hand-washing, and social distancing, which help protect both citizens themselves and fellow citizens from exposure to the virus [9]. Moreover, many governments enforced these guidelines under the threat of punishment in national emergencies or lockdowns. While efforts to create effective treatments and vaccinations have progressed, illness control still relies on modifying citizens’ behavioral patterns in order to reduce the ability of the virus to spread. Thus, the outcome of such illness control is contingent upon whether citizens adhere to preventive behavior norms and advice [10]. While vaccinations protect against severe sickness, hospitalizations, and deaths caused by the early SARS-CoV-2 variant, it remains uncertain how effective the vaccines will be against future variants [11]. In the absence of effective treatments, worldwide vaccine availability, or widespread herd immunity, COVID-19 prevention behaviors are much more effective when they are done collectively [8, 12, 13]. Still, many citizens failed to adopt such disease prevention behaviors, which made it difficult for governments to contain the spread of the virus [14]. Our study aims to examine the reasons behind interpersonal differences in the adoption of these recommended disease prevention behaviors.

**Fig. 1:**
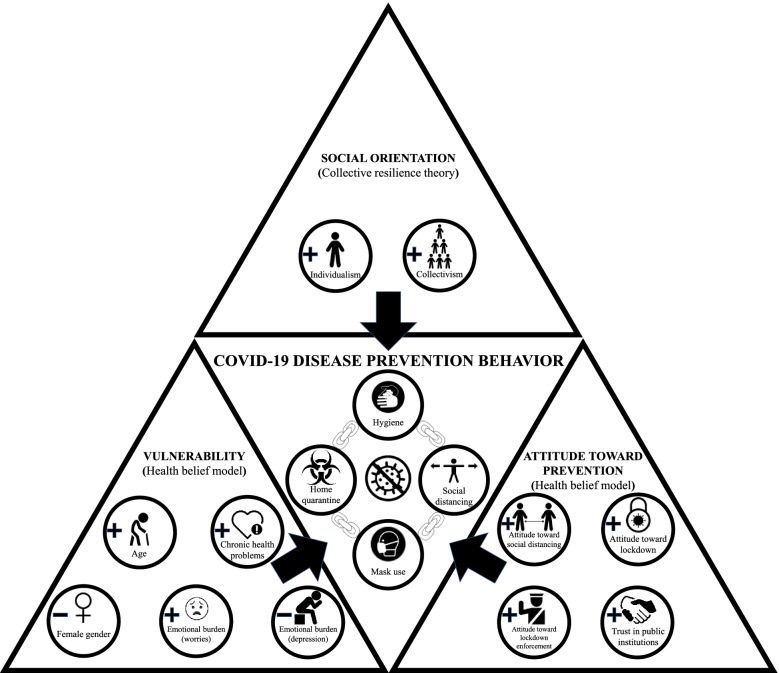
Graphical abstract

In the literature on disease prevention behavior, the COVID-19 pandemic stands out in two ways. First, the recommended disease prevention behaviors were issued to protect not only the citizens themselves, but also their fellow citizens [9]. Therefore, these behaviors may be motivated not only by self-protection, which is the focus of most theories of disease prevention behavior (e.g., the health belief model: Rosenstock [15]), but also by the selfless protection of their fellow citizens. Second, most societies treat the COVID-19 pandemic as a national disaster rather than as an individual problem. This part is evidenced by the leadership of governments, by daily media coverage, and significant changes that permeated societies, such as national lockdowns, large-scale telework, and the visible omnipresence of people wearing masks [16, 17]. As predicted by collective resilience theory, national disasters can reinforce the willingness to engage in selfless acts to protect fellow citizens, which we posit to apply equally to disease prevention behaviors that protect fellow citizens [18]. Therefore, we predict that the reasons for COVID-19 disease prevention behavior consist of both self-preservative motives, which we explain based on the health belief model [15], and selfless motives to protect fellow citizens, which we describe based on collective resilience theory [18] (see graphical abstract in Fig. 1). This focus on collective resilience differentiates our study from the recent literature on COVID-19 prevention behavior, which almost exclusively focuses on self-preservation (e.g., Kwok et al. [19]; Shahnazi et al. [20]. We test our hypotheses through hierarchical linear modeling of data from 1231 citizens.

Consequently, we make two original contributions to the literature based on the study results. First, the nature of the COVID-19 pandemic as a national calamity motivates citizens with a specific social orientation to adopt disease prevention behaviors. This result is a unique contribution of our study based on collective resilience theory. Second, we find a non-linear relationship between chronic health problems and disease preventive behavior based on the health belief model. We also find a strong association between lockdown-related attitudes and disease prevention behavior. In the following sections, we develop these contributions theoretically and test them empirically. Eventually, we propose measures to increase the adoption of COVID-19 prevention behaviors.

## Conceptual background

### The literature on a Citizen’s individual disease prevention behavior

The field of public health encompasses areas such as preventing diseases and death, promoting a better quality of life, and creating healthy environments. The effectiveness of public health policies and recommendations depends on the accurate identification and definition of public health problems, the determination of populations at risk, the understanding of the factors driving an individual’s healthy behaviors, the development and implementation of evidence-based interventions, and the continuous evaluation of such interventions [21]. A literature review suggests that scholars mainly draw on eight theories to understand the psychological process that motivates individuals to pursue healthy behaviors, such as disease prevention. These are the health belief model (Rosenstock [15]; meta-analysis by Carpenter [22]), the theory of planned behavior (Ajzen [23]; meta-analysis by Hagger et al. [24]), the theory of diffusion of innovations [25], social cognitive theory (Bandura [26]; meta-analysis by Zhang et al. [27]), the transtheoretical model [28, 29], social norms theory [30], behavioral economics [31], and protection motivation theory [32]. For our research on disease prevention behavior in the context of COVID-19, we adopt the health belief model, which is the model most specific to disease prevention behavior and thus best at predicting such behavior [22]. Moreover, while other models (e.g., social cognitive theory) with a dynamic perspective focused on longitudinal changes over time leverage their predictive strength in a retrospective analysis of recurrent health behaviors, the health belief model has a static perspective and allows for early predictions in a novel public health crisis [22, 27].

### The health belief model

Proposed by Rosenstock [15], the health belief model posits that five variables influence an individual’s pursuit of healthy behaviors, such as disease prevention behavior, and can thus be targeted by communication campaigns to improve public health: susceptibility, severity, benefits, barriers, and cues. Susceptibility refers to a person’s belief about the likelihood of being affected by a negative health consequence (e.g., COVID-19). Severity refers to the belief that this negative health consequence has an adverse impact on the person’s life (i.e., death or long-term health damage from COVID-19, rather than recovery). Benefits refer to the belief that a certain target behavior (e.g., social distancing) helps prevent or treat the negative health consequences. Barriers are the belief that the person cannot easily adopt a target behavior (e.g., too expensive, painful, or challenging). Finally, cues refer to stimuli that trigger the motivation to engage in disease prevention behavior, such as a media campaign (external cue) or a deteriorating body shape (internal cue). As Rosenstock’s [15] descriptions of cues are less developed, most studies on the health belief model exclude this variable [22, 33]. In a meta-analysis excluding cues, Carpenter [22] finds that benefits have the strongest association with disease prevention behavior (*r =* .42), followed by barriers (*r =* .33), severity (*r =* .16), and susceptibility (*r =* −.06, mostly non-significant).

Consequently, in using the health belief model as a theoretical lens to explain disease prevention behavior in the context of COVID-19, we regard public health recommendations [1, 9] and legal restrictions on citizens’ behaviors in the form of a national or regional lockdown [17] as the principal cues affecting all citizens equally. Moreover, we adopt the view that individual differences in beliefs about benefits, severity, and barriers are the leading causes of individual differences in citizens’ disease prevention behaviors and thus in their compliance with guidelines.

### Extending the health belief model: collective resilience of a society to diseases

The health belief model and the other theories of disease prevention behavior focus on an individual’s behavior, such as the avoidance of drugs or precautions against obesity [22]. However, the COVID-19 pandemic exceeds the scope of an individual’s behavior. It triggers a national movement as evidenced by organized national campaigns to combat this crisis, daily coverage on TV channels, and restrictions on economic, social, and individual activity unseen in many democratic countries since the Second World War [16, 17, 34]. Moreover, governments and the WHO [1] promote health prevention behaviors not only for self-protection but also for protecting fellow citizens and society from coronavirus infections. Hence, the COVID-19 pandemic is not merely a matter of individual self-protection but also a case of a collective response to a national disaster.

The literature reports that national disasters trigger self-preservation and selfless and sometimes risky actions to protect and aid fellow citizens [18]. Such actions occur both in the immediate locale of a disaster (e.g., rescuing strangers after an earthquake) and in locations far removed from a disaster, as evidenced by intentional consumer purchases of food products grown in a radioactively contaminated area after the accident at the Fukushima nuclear power plant [35, 36]. Consequently, we predict that recognizing the COVID-19 pandemic as a national disaster may trigger a citizen’s intention to engage in disease prevention behaviors not only to protect oneself, but also to protect fellow citizens. From a medical perspective, due to the long incubation period of the coronavirus (2 to 14 days), individuals may be infected and pass on the coronavirus without experiencing any symptoms [1, 37, 38]. Thus, citizens may pursue disease prevention behaviors such as social distancing, a home quarantine, hygiene, and mask use to protect both themselves and other fellow citizens and society.

Among multiple theories that describe selfless acts during national disasters, we draw on the collective resilience theory [18] because it does not require the focal person to be present at a location with visible victims. Instead, it operates through cognitive processes that function in areas removed from visible victims [35, 36], such as at home without COVID-19 victims. Collective resilience theory [18] builds upon self-categorization theory [39]. It predicts that a salient self-categorization as a potential victim enhances an individual’s motivation to engage in self-preservative behavior. In contrast, a salient self-categorization as a collective member perceived as threatened by a national disaster enhances the individual’s motivation to engage in the selfless support of fellow members of this collective. For the context of the COVID-19 pandemic, we thus predict that both a salient self-categorization as a potential victim and a salient self-categorization as a member of a collective (e.g., society) threatened by the pandemic may motivate a person to pursue disease prevention behaviors aimed at both self-preservation and protection of others. Consequently, we aim to extend the health belief model by a set of variables related to the salience of individual and collective self-categorizations in order to enable the model to explain a citizen’s disease prevention behavior in a pandemic recognized as a national disaster.

### Conceptual model and selection of variables

**Fig. 2:**
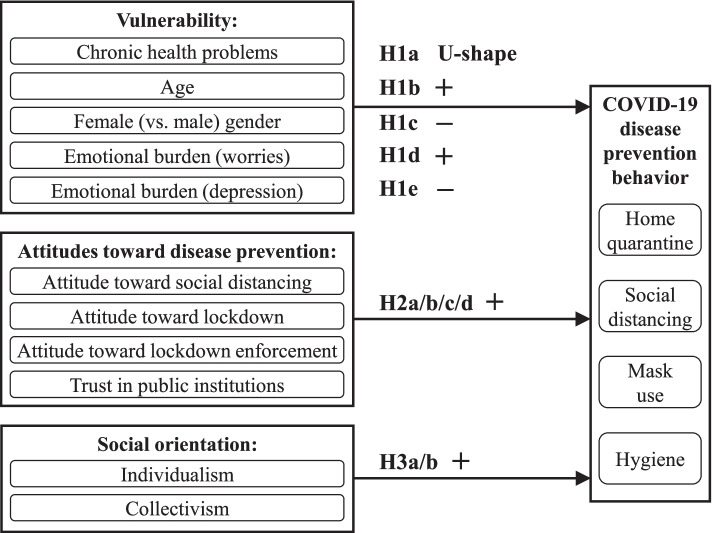
Conceptual framework and hypotheses

Based on our theory development, we construct a conceptual model (see Fig. 2) predicting disease prevention behavior in a national crisis (e.g., the COVID-19 pandemic) by a person’s vulnerability (hypotheses H1a-e), attitudes toward disease prevention (H2a-d), and social orientation (H3a/b). These categories encompass the predictors discussed in the health belief model and collective resilience theory.

In the health belief model [15], a person’s vulnerability corresponds to beliefs about the severity of adverse health consequences and barriers to adopting a disease prevention behavior. That is, physical or psychological constraints may increase the likelihood of death from COVID-19 [1, 38], but they also make it more challenging to adopt disease prevention behaviors, such as when hospital visits to treat chronic health problems interfere with a home quarantine or social distancing. Moreover, in the health belief model, a person’s attitudes toward disease prevention correspond to beliefs about the benefits of adopting disease prevention behaviors for avoiding a negative health consequence. Extending the health belief model, a person’s social orientation refers to how a person views social relationships and balances individual interests with those of the larger group to which the person belongs [40]. Social orientation may be particularly relevant in the context of a national pandemic, where an individual seeks to balance his or her interests with the interests of society. Through the lens of the health belief model, social orientation can be understood as resulting in another social type of psychological barrier to individual action, such as when people believe that their actions do not determine their fate (i.e., low horizontal individualism [40] and thus do not protect themselves individually.

In collective resilience theory [18], a person’s vulnerability may cause self-categorization as a potential victim of disease to become salient and may thus trigger self-preservative behaviors. A positive attitude toward disease prevention behaviors recommended by the government to combat a national crisis (e.g., toward the enforcement of a home quarantine) may reflect a salient self-categorization as a member of society during a national disaster. According to collective resilience theory, this would trigger health prevention behavior not only for self-protection but also for protecting fellow citizens and society [18, 36]. Moreover, a person’s social orientation may reflect the tendency for self-categorization at the individual or collective level, which would affect disease prevention behavior for individual or social motives [35].

While the health belief model thus guides our selection of variables to explain a person’s disease prevention behavior for self-preservative motives, collective resilience may strengthen those disease prevention behaviors that also benefit fellow citizens. This aspect provides compelling reasons for including social orientation in our conceptual model (see Fig. 2), which predicts behavior in a national pandemic.

### The literature on COVID-19 disease prevention behavior

Table 1 summarizes the literature on disease prevention behavior in the context of COVID-19. Most studies illuminate how certain aspects of a person’s vulnerability, such as the health risk (gender, age, location with many infections), psychological weakness (anxiety), lack of knowledge (information, education), and perceived barriers in the adoption of behavior, are associated with COVID-19 prevention behavior. A few studies also explore how attitudes toward disease prevention, such as the perceived benefits of disease prevention behavior and trust in the received information about disease prevention are associated with COVID-19 prevention behavior. By contrast, few studies examine the associations between social orientation and COVID-19 prevention behavior. These studies explore the roles of empathy toward fellow citizens, perceived social pressure to engage in disease prevention behavior, and political and ethical values, which partially relate to beliefs of how fellow citizens should treat each other.

**Table 1:** The literature on the determinants of a person’s COVID-19 disease prevention behavior

						Effects of independent variables		
Year	Authors	Theory	Country	Sample size	Dependent variable	Effects of vulnerability	Effects of attitudes toward disease prevention	Effects of social orientation
2020	Alzoubi et al.	-	Jordan	592 students	Disease prevention behavior	Education type (medical vs. non-medical colleges) (n.s.)	-	-
2020	Bashirian et al.	Protection motivation theory	Iran	761	Disease prevention behavior	Threat appraisal (susceptibility + severity) (+)	Coping appraisal (feasibility + benefits - costs of prevention behavior) (+)	-
2020	Chang et al.	-	Taiwan	414 patients	Disease prevention behavior	Fear of disease (-), psychological distress (n.s.), self-stigma (n.s.)	Trust in information about disease prevention (+)	-
2020	Chen et al.	-	China	8569 students	Hand-washing / mask-wearing	Local spread of disease (+/+), female gender (+/n.s.), education (+/+), parents' education (-/+), out-going history (+/not tested)	-	-
2020	Chen and Chen	Theory of reasoned action	China	1591	Disease prevention behavior	Rural residence (n.s.)	Perceived benefits of disease prevention behavior (+), information appraisal (+)	Subjective norms (+)
2020	Everett et al. (no peer review)	-	U.S.	1032	Disease prevention intentions	Age (+), female gender (+), white ethnicity (-), education (-), income (n.s.), employment (n.s.)	-	Political conservatism (-), religiosity (+)
2020	Harper et al.	Moral foundations theory	U.K.	324	Increase in disease prevention behavior	Fear (+), depression (-), anxiety (n.s.), perceived risk (+)	-	Political orientation (n.s.), moral standards (n.s.)
2020	Lee and You	Risk perception attitude framework	South Korea	973	Disease prevention behaviors	Age (+), female gender (+), education (+), income (+), city residence (-), presence of children (+), subjective health (+), perceived susceptibility (n.s.), perceived severity (+), social support (+)	Perceived benefits of disease prevention behavior (+)	-
2020a	Li et al.	Cognitive appraisal theory	China	4607	Disease prevention behavior	Age (-), female gender (+), education (+), psychological problems (n.s.), chronic disease (+), health condition (+), sick relatives (n.s.), knowledge (+), perceived severity (+)	Perceived controllability (+)	-
2020b	Li et al.	-	U.S.	979	Disease prevention behavior	Age (+), female gender (+), white ethnicity (-), marriage (+/-), income (+), education (-), employment (-), knowledge (+), susceptability (+)	-	-
2020	Min et al.		China	3000	Disease prevention behaviors	Age (n.s.), female gender (n.s.), education (n.s.), marital status (+), city residence (n.s.), income (+), knowledge (+), negative emotion (n.s.)	Trust in public institutions (+)	
2020	Kwok et al.	Health belief model	Hong Kong (China)	1715	Social distancing	Age (n.s.), female gender (+), disease knowledge (+), visits to China (+), residence near border to China (+), chronic diseases (n.s.), anxiety (+)	-	-
2020	Oosterhoff et al.	-	U.S.	683 adolescents	Social distancing	Age (n.s.), female gender (n.s.), white / hispanic ethnicity (-), financial strain (n.s.), parents' education (+), lockdown (+), parents' rules (+)	Importance of self-protection (n.s.), perceived lack of alternatives (+), preference to stay home (n.s.)	Social pressure (n.s.), social responsibility (+), importance of protecting others (n.s.)
2020	Pfattheicher et al.	Prosocial behavior	U.S., U.K., Germany	3718	Social distancing / Mask-wearing	-	-	Empathy (+)
2020	Prasetyo et al.	Protection motivation theory	Philippines	649	Disease prevention behavior	Understanding of disease (+), perceived severity (+), perceived vulnerability (-), anxiety (+)	Perceived behavioral control (+)	Subjective norm (+)
2020	Shahnazi et al.	Health belief model	Iran	750	Disease prevention behavior	Age (n.s.), female gender (+), rural residence (+), barriers (-), susceptibility (n.s.), severity (n.s.), self-efficacy (+), disease syndromes (n.s.)	Perceived benefits of disease prevention behavior (+), fatalistic beliefs (-)	-
2020	Taghrir et al.	-	Iran	240 students	Disease prevention behavior	Disease knowledge (n.s.), perceived risk (-)	-	-
2020	Yıldırım et al.	-	Turkey	3190	Disease prevention behavior	Age (n.s.), female gender (+), severity (+), self-efficacy (+), knowledge (n.s.), mental health (+)	-	-
2021	Bronfman et al.	-	Chile	1004	Disease prevention behavior	Female gender (+), family size (-), income (-)	Trust in government (+)	-
2021	Ezati-Rad et al.	Protection motivation theory	Iran	2032	Disease prevention behavior	Threat apraisal (+), fear of disease (+)	Motivation (+), coping appraisal (+), maladaptive behavior rewards (-), perceived costs (-)	-
2021	Firouzbakht et al.	-	Iran	2097	Disease prevention behavior	Female gender (+), age (+), education (+), income (+)	Attitude toward face mask and gloves use (+)	-
2021	Guo et al.	E-health literacy	Hong Kong (China)	1501	eHealth literacy score	Older age (-), female gender (n.s.), marital status (n.s.), education (+), high income (+), employment (n.s.), chronic disease (n.s.)	-	-
2021	Hosen et al.		Bangladesh	10067	Disease prevention behavior	Age (n.s.), female gender (+), employment (+), divorced/widowed (-), rural residence (-), education (-), knowledge (+), alcohol consumption (-), smoking (-)	-	-
2021	Šuriņa et al.	Protection motivation theory	Latvia	2606	Disease prevention behavior	Fear of disease (+), threat appraisal (+)	Conspiracy beliefs (n.s.), trust in information sources (+)	-
2021	Yıldırım et al.	-	Turkey	4536	Disease prevention behavior	Age (+), female gender (+), education (+), vulnerability (+), perceived risk (+), fear (+)	-	-
2022	This article	Health belief model, collective resilience theory	Bolivia	1231	Disease prevention behavior	Age (+), female gender (+), education (n.s.), climate (n.s.), income-oriented work (n.s.), population density (n.s.), chronic health problems (U-shaped effect), depression (-), worries (+)	Attitude toward social distancing (+), attitude toward lockdown (+), attitude toward lockdown enforcement (+), trust in public institutions (+)	Individualism (+), collectivism (+)

With our conceptualization of COVID-19 prevention behavior as partially reflecting collective resilience in a national disaster, we extend this literature by examining how hitherto unexplored types of social orientation are associated with COVID-19 prevention behavior. In addition, we explore the associations between COVID-19 prevention behavior and those attitudes toward disease prevention that concern compliance with the society’s collective approach to containing the disease. Moreover, we explore the associations between COVID-19 prevention behavior and additional aspects of a person’s physical and psychological vulnerability resulting from the society’s collective response to the pandemic, such as the physical, psychological, and income-related vulnerability caused by the constraints of a national lockdown.

## Development of hypotheses

### The association between vulnerability and disease prevention behavior

Concerning a person’s vulnerability in the context of COVID-19, we focus on the main physiological risk factors (male gender, high age, chronic health problems [1, 38]), and on psychological vulnerability (worries, depression) caused by the pandemic. According to the health belief model, susceptibility and severity as critical aspects of a person’s vulnerability have positive associations with the adoption of disease prevention behavior, whereas barriers have a negative association with it [15].

First, the health belief model predicts that both the perceived susceptibility to contracting a disease and the expected severity of an acquired disease increase the perceived health risk and thus alert the person to engage in disease prevention behavior to limit the elevated health risk [12, 15, 41–43]. The perception of susceptibility to contracting the coronavirus is higher for individuals who worry about their infection risk. Such worries may result from extensive media coverage of the pandemic as a national disaster [17]. Moreover, as broadly disseminated by the media, the severity of COVID-19 is higher for people with increased age, male gender, and chronic health problems [1, 37, 44]. Consequently, the health belief model would suggest that worries, age, male gender, and chronic health problems are positively associated with a person’s COVID-19 disease prevention behavior.

Second, the health belief model predicts that perceived barriers to disease prevention reduce the perceived ability to adopt disease prevention behaviors (i.e., self-efficacy), which affects such behaviors negatively [15]. In particular, high levels of fear over a long period during a health crisis accumulate into severe mental health problems [6]. From this perspective, depression, which may result from negative media coverage and the behavioral constraints of a lockdown as the collective response to the pandemic as a national disaster [6, 45, 46], may reflect helplessness and low self-efficacy [47] and thus the belief of being unable to cope with the threat of disease. Hence, we posit that depression is negatively associated with disease prevention behavior. In addition, many people with chronic health problems undergo frequent health treatment, which requires them to visit medical facilities. These visits increase their exposure to a disease, such as COVID-19, and constitute a barrier to adopting disease prevention behavior (e.g., social distancing). According to the health belief model, the barrier resulting from the treatment of chronic health problems would reduce disease prevention behavior, whereas the higher severity of COVID-19 would increase it. Given the trade-off resulting from these opposing mechanisms, we predict that people with minor chronic health problems and only a slightly elevated COVID-19 death risk [1, 37] would still seek regular medical treatment and would thus limit their disease prevention behavior. By contrast, people with severe chronic health problems and a very high COVID-19 death risk [1, 37] may prioritize disease prevention and thus create larger intervals in seeking medical treatment. The previous arguments suggest that chronic health problems have a U-shaped association with disease prevention behavior.

**H1a:** Chronic health problems have a U-shaped association on disease prevention behavior.

**H1b:** Age is positively associated with disease prevention behavior.

**H1c:** Compared with women, men engage in more disease prevention behavior.

**H1d:** Worries are positively associated with disease prevention behavior.

**H1e:** Depression is negatively associated with disease prevention behavior.

### The associations between attitudes toward disease prevention and disease prevention behavior

A person’s attitudes toward disease prevention behavior reflect whether a person believes in the benefits of such behavior for avoiding a negative health consequence. The health belief model predicts that these beliefs are positively associated with disease prevention behavior [15]. In most countries, the COVID-19 pandemic has gained recognition as a national disaster, which has prompted governments to take the lead in coordinating a collective response to the pandemic in the form of behavioral guidelines for disease prevention behavior and legal measures to impose such behavior [17]. Therefore, we focus on beliefs about the benefits of government-initiated guidelines and initiatives.

During the onset of the COVID-19 pandemic, most Western governments emphasized social distancing as the premier way of containing the spread of COVID-19 and imposed a strict lockdown [17]. Hence, based on the health belief model, we predict that a person’s attitudes toward social distancing, a government-imposed lockdown, and the enforcement of this lockdown are positively associated with disease prevention behavior during this phase of the pandemic. Moreover, since government institutions took the lead in communicating behavioral guidelines [9, 37, 48, 49], we predict that a person’s trust in public institutions determines the degree to which the person believes in the benefits of these guidelines and complies with them. This prediction follows the source credibility model, which emphasizes the role of trust in absorbing information from a source [50].

While the health belief model focuses on a person’s motivation to protect oneself, COVID-19 disease prevention behavior (e.g., social distancing, hygiene, and mask use) benefits not only oneself but also other members of society [9]. To explain this additional mechanism, we draw on collective resilience theory [18], which predicts that a salient self-categorization as a member of a collective perceived as threatened by a national disaster enhances the person’s motivation to protect fellow members of this collective. We assume that the pivotal role of the government in communicating guidelines on disease prevention and enforcing a lockdown enhances the salience of most citizens’ self-categorization with the society threatened by COVID-19. This salient collective self-categorization may reinforce a person’s tendency to comply with government guidelines on social distancing, a lockdown, and its enforcement to prevent fellow citizens from being infected. Moreover, the preference for such selfless support of fellow citizens is likely larger when a person trusts the government, which may enhance the salience of this person’s self-categorization with the threatened society.

**H2a:** The attitude toward social distancing is positively associated with disease prevention behavior.

**H2b:** The attitude toward a government-imposed lockdown is positively associated with disease prevention behavior.

**H2c:** The attitude toward the government’s enforcement of a lockdown is positively associated with disease prevention behavior.

**H2d:** The trust in public institutions is positively associated with disease prevention behavior.

### The association between social orientation and disease prevention behavior

Given the recognition of the COVID-19 pandemic as a national disaster that threatens society, we also focus on variables related to a person’s social orientation, which we define as an inherent tendency for self-categorization at the individual or collective level. Hofstede’s model conceptualizes such social orientation on a scale of individualism versus collectivism, which reflects the prioritization of individual versus collective interests in decisions [51]. Since Hofstede’s measure does not satisfy psychometric requirements, such as convergent and discriminant validity, the measurement of individualism and collectivism as separate dimensions, sometimes even with subdimensions [40], has become the dominant approach in cultural psychology [52]. Individualism reflects a self-categorization (i.e., a definition of oneself) as an individual that is independent from any groups and autonomous in decisions on his or her own actions. It reinforces the pursuit of personal rather than group-related goals. Collectivism reflects a self-categorization as a group member, such as society. It results in prioritizing group goals over individual goals [40]. In this model, individualism and collectivism are not strict opposites because an individualistic person may belong to groups and consider them important [52].

Examined through the theoretical lens of the health belief model, social orientation may alter the psychological barriers to disease prevention. An individualistic orientation reinforces a person’s pursuit of personal goals [52], such as disease prevention, and may thus weaken psychological barriers to action, resulting in more disease prevention behavior. However, as the health belief model focuses on individual disease prevention, it cannot predict the consequences of a collectivist orientation. Therefore, we draw on collective resilience theory, which states that a salient self-categorization with a collective threatened by a national disaster, such as the COVID-19 pandemic [17], results in selfless actions to support members of this collective [18]. Resulting from this salient self-categorization with groups (society, family, or friends) threatened by COVID-19, a person may adopt disease prevention behaviors, such as social distancing or mask use, to protect others from oneself as a potential source of infection. We posit that this tendency is stronger for a person with a collective orientation that prioritizes the interests of certain groups. In line with this prediction, protests against mask use and social distancing as measures to stop people from unknowingly spreading a COVID-19 infection to others [14, 53] occurred primarily in countries with lower collectivism [52, 54].

**H3a:** An individualistic orientation is positively associated with disease prevention behavior.

**H3b:** A collectivist orientation is positively associated with disease prevention behavior.

## Method

### Measures

To test our hypotheses, we designed a questionnaire survey based on multi-item scales. The Additional file 1 lists all scales, the wording of their measurement items, and their literature sources. We obtained the scales related to disease prevention behavior in the past week, most attitudes toward disease prevention, and vulnerability (chronic diseases and emotional burden during the past 2 weeks) from [55]. However, we revised and appended these scales based on the latest disease prevention guidelines from the WHO [1] and CDC [9]. We measured trust in public institutions with a scale from Listhaug and Ringdal [56] and individualistic and collectivist orientation with the scale of horizontal and vertical individualism and collectivism from Singelis et al. [40]. Moreover, as control variables and their components, we measured the respondent’s level of education (1: below high school; 2: high school; 3: bachelor’s degree; 4: post-graduate education below master’s level; 5: master’s degree; 6: Ph.D. degree), occupation, and region of birth. This approach reflects that a citizen’s disease prevention behavior might result from the ability to process information, work-related obligations, and childhood experiences of disease prevention behavior tied to climatic conditions and population density.

We targeted the questionnaire at respondents in Bolivia and thus prepared a Spanish language version. In early 2020, Bolivia experienced an exponential increase in coronavirus infections, similar to most countries [38]. A nationwide quarantine began on March 22, 2020, and was upheld throughout the period of data collection [17].

### Sample

Using an anonymous online survey, we collected data in Bolivia from March 24 to April 4. In distributing the link across the entire country, we received support from government officials, priests of the Catholic Church (77% of the population Catholic [57]), and professors spread across the country. Based on the distribution of IPs, people accessed the survey from all across Bolivia. Among 5265 persons who accessed the questionnaire, 1857 partially filled it out. After removing data with missing responses to any variable, the final sample size is 1231 respondents. 57% of the respondents are female. The mean age is 31 years, and the median age is 26 years. This result is slightly older than the Bolivian median age of 25.6 years (Worldometer [58], based on UN data) and means that the age-related risk of death from COVID-19 is lower than in countries with elderly populations [38]. Table 2 shows the descriptive statistics and correlations of the variables. These statistics indicate a high reported compliance with the government’s home quarantine order and hygiene recommendations, and a positive attitude toward these government measures, despite low trust in public institutions [59].

**Table 2:** Correlations and descriptive statistics of multi-item measures

	Correlations										
Variables	1	2	3	4	5	6	7	8	9	10	11
*First-order constructs:*											
1 Attitude toward social distancing											
2 Attitude toward lockdown	.32										
3 Trust in public institutions	-.03	.07									
4 Horizontal individualism	.05	.09	-.04								
5 Vertical individualism	.03	.02	.15	.15							
6 Horizontal collectivism	.15	.14	-.03	.25	.02						
7 Emotional burden (worries)	.18	.22	.05	.02	.11	.05					
8 Emotional burden (depression)	-.03	.02	.09	-.04	.08	-.13	.36				
9 Chronic health problems	.02	.01	.01	-.09	-.02	-.03	.22	.26			
*Second-order constructs:*											
10 Disease prevention behavior	.30	.26	.05	.12	.09	.19	.13	-.08	-.02		
11 Attitude toward lockdown enforcement	.22	.40	.01	.04	.10	.11	.23	.03	-.02	.25	
*Descriptive statistics:*											
Mean	9.60	8.93	3.04	8.44	5.84	8.97	6.38	3.88	.00	8.46	8.40
Standard deviation	1.15	1.83	1.70	1.53	2.56	1.26	2.32	2.29	.79	1.31	1.83
Composite reliability	.88	.70	.90	.75	.86	.80	.78	.89	formative measures (see appendix)		
Cronbach's α	.86	.68	.89	.75	.86	.79	.78	.89			
Average variance extracted	.70	.54	.59	.60	.68	.67	.54	.55			
Square root (average variance extracted)	.84	.73	.77	.78	.82	.82	.74	.74			
Number of scale items	3	2	6	2	3	3	3	7	2	10	9

All correlations |r| ≥ .07 are significant at *p* < .05 (two-sided). Sample size: 1231. Descriptive statistics for mean score across non-standardized items (except for variable 9: standardized items due to different units per item)

### Data validity

#### Convergent and discriminant validity

To measure the convergent and discriminant validity of the reflective multi-item scales, we performed a confirmatory factor analysis that resulted in the following fit measures: χ^2^/df = 2.71, CFI = .95, and RMSEA = .04, and upper bound of 90% RMSEA confidence interval = .04. These fit measures satisfy the common acceptance criteria (χ^2^/df < 5, CFI ≥ .95, RMSEA ≤ .07, upper bound of 90% RMSEA confidence interval ≤ .1; Hair et al. [60]). Moreover, as summarized in Table 1 and the Additional file 1, the reflective constructs of our survey meet the common requirements of convergent and discriminant validity (composite reliability > .7, average variance extracted > .5, and average variance extracted > squared correlations with other constructs; Hair et al. [60]).

#### Common method variance (CMV)

To minimize a bias in our results due to CMV, our dependent variable of disease prevention behavior focuses on actual behavior rather than future intentions. Moreover, we measured this variable before all other variables to prevent CMV bias and priming effects from reading the questions belonging to our independent variables. For estimating the extent of CMV in the collected data, Lindell and Whitney [61] recommend interpreting as an upper bound on CMV the second-smallest positive correlation between all variables measured by Likert scales in a dataset. In our correlation matrix (see Table 2), this correlation is minimal (.02) and non-significant. Hence, CMV is unlikely to threaten the validity of the conclusions drawn from our data.

## Results

### Hypothesis tests

#### Model structure

To test our hypotheses, we used hierarchical linear modeling (HLM) to account for the potential clustering of our data in the geographically and racially diverse country of Bolivia. Our cross-classified HLM model treats respondents at level 1, regions of birth (9 official regions; foreign countries for immigrants) at level 2a, and occupations (9 categories) at level 2b. As control variables at level 2a, our HLM model includes the population density and the dominant climate zone (1: polar; 2: temperate; 3: tropical) in the region of birth. These variables shape the contagiousness of viruses such as seasonal influenza or coronaviruses [62], which may have affected childhood experiences in disease prevention behavior with a potentially lasting effect on current practices. At level 2b, our HLM model controls whether the respondent currently pursues income-oriented work (1: working with income; 0: otherwise). Such work may affect disease prevention behavior by making hygiene difficult in occupations without regular access to water and soap or making social distancing a potential threat to the ability to survive financially in Bolivia as one of the poorest countries of the Americas [57]. At level 1, our HLM model controls for the respondent’s level of education to account for potential differences in understanding the relevance of prioritizing disease prevention behavior over social experiences and work in a low-income country. The HLM model also contains an intercept and error terms at levels 1, 2a, and 2b. Disease prevention behavior in the past week serves as the dependent variable. Moreover, the HLM model includes all independent variables in the focus of our hypotheses. We standardized all variables in the analysis because we used different scale units to measure them. Table 3 presents the results of our study. Since all variance inflation factors in the HLM model are below two, multi-collinearity is not a concern [63]. The pseudo R^2^ measure indicates that our model explains only 20% of the variance in reported disease prevention behavior. While the explanatory power of statistical models describing actual, rather than intended, behavior generally tends to be lower as a myriad of situational circumstances may interfere with intentions [64], there appear to be other predictors that our model does not capture.

**Table 3:** The association between personal characteristics and COVID-19 disease prevention behavior

Independent variables	β
*Control variables:*	
Intercept	-.233
Education	.001
Dominant climate in region of birth (1: polar; 2: temperate; 3: tropical) (level 2a)	.033
Population density in region of birth (level 2a)	.021
Income-oriented work (1: working with income; 0: otherwise) (level 2b)	-.041
*Vulnerability:*	
Chronic health problems	-.073*
(Chronic health problems)^2^ (H1a: +)	.026*
Age (H1b: +)	.131***
Female (vs. male) gender (1: female; 0: male) (H1c: -)	.092***
Emotional burden (worries) (H1d: +)	.055†
Emotional burden (depression) (H1e: -)	-.076*
*Attitudes toward disease prevention:*	
Attitude toward social distancing (H2a: +)	.187***
Attitude toward lockdown (H2b: +)	.106***
Attitude toward lockdown enforcement (H2c: +)	.141***
Trust in public institutions (H2d: +)	.055*
*Social orientation:*	
Horizontal individualism (belief in self-determined fate) (H3a: +)	.059*
Vertical individualism (belief in competition with others) (H3a: +)	.062*
Horizontal collectivism (belief in helping others) (H3b: +)	.077**
*Covariance parameters:*	
Residual at level 1 (person)	.799***
Residual at level 2a (region of birth)	.547
Residual at level 2b (occupation)	.001
*Fit statistics:*	
HLM pseudo R^2^	.201
Sample size	1231

†*p* < .1; *p < .05; **p < .01; ***p < .001 (two-sided *p*-values). Effects of standardized variables. Cross-classified hierarchical linear modeling (HLM) (level 1: person; level 2a: region; level 2b: occupation)

#### Tests of hypothesized associations

**Fig. 3:**
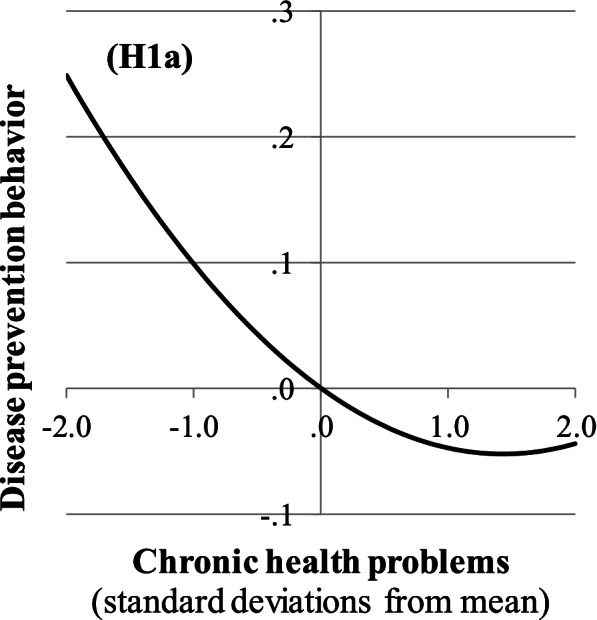
The non-linear association between chronic health problems and disease prevention behavior

Regarding a citizen’s vulnerability, chronic health problems have a U-shaped association with disease prevention behavior (H1a supported). As shown in Fig. 3, the degree of chronic health problems that minimizes disease prevention behavior is relatively high, and both more and fewer chronic health problems are associated with more disease prevention behavior. Moreover, women and higher-aged citizens engage in significantly more disease prevention behavior than men and younger citizens (H1b supported, H1c not supported). Concerning the emotional burden of the coronavirus crisis and the lockdown, worries have a positive association with disease prevention behavior (H1d marginally supported at two-sided *p* < .1, corresponding to one-sided *p* < .05 for our one-sided hypothesis). In contrast, depression has a negative association with disease prevention behavior (H1e supported).

Regarding attitudes toward disease prevention, trust in public institutions, and attitudes toward social distancing, a government-imposed lockdown, and the enforcement of this lockdown have positive associations with disease prevention behavior (H2a/b/c/d supported). Regarding social orientation, we adopted the multi-dimensional view of individualism and collectivism from Singelis et al. [40]. Horizontal individualism (belief in self-determined fate), vertical individualism (belief in competition with others), and horizontal collectivism (belief in helping others) have positive associations with disease prevention behavior (H3a/b supported). In summary, the results support our hypotheses. A comparison of nominal effect sizes indicates that age and attitudes toward disease prevention have the strongest associations with disease prevention behavior.

### Robustness tests and additional analyses

#### Squared terms

When including additional squared terms of all variables, we find that the squared terms of attitude toward lockdown enforcement and attitude toward social distancing are positively associated with disease prevention behavior. This indicates that the hypothesized associations between these independent variables and the disease prevention behavior actually have slightly increasing marginal returns.

#### Vertical individualism

Our survey also measures vertical collectivism (belief in the duty to support family members; Singelis [40]), which equally affects disease prevention behavior positively (in line with H3b). However, this measure does not satisfy the criteria of convergent validity [60].

#### Hygiene to protect only others, not oneself

While our dependent variable of disaster prevention behavior refers to behaviors that protect both the respondents themselves and other members of society, our survey includes an alternative single-item variable concerning behaviors only benefitting other members of society, but not the respondents themselves. We developed this item based on recommendations by the CDC [9] and WHO [1]: “During the last week, when I sneezed or coughed, I covered my mouth and nose with my flexed elbow or with a napkin.” We obtained the following results using this variable as an alternative dependent variable [40]. None of the variables related to vulnerability have a significant association with this dependent variable. Only the respondent’s attitudes toward social distancing and enforcement of the lockdown, horizontal individualism, and horizontal (and vertical) collectivism are related to this particular type of disaster prevention behavior, which benefits only other citizens. The associations between collectivism and this dependent variable become much higher than in the main hypothesis test of Table 2 (β = .17, *p* < .001).

## Discussion

### Theoretical implications

For the context of the COVID-19 pandemic, our study examines the reasons for interpersonal differences in adopting disease prevention behaviors that protect both citizens themselves and other fellow citizens [9]. Our study makes two contributions to the literature.

First, we extend the health belief model [15] to explain disease prevention behavior. Based on this theory, we focus on variables related to a citizen’s vulnerability and attitudes toward disease prevention behavior. In the terminology of this theory, vulnerability comprises beliefs regarding one’s susceptibility to the disease, the severity of the disease, and barriers in the adoption of disease prevention behavior [15]. COVID-19 being more severe in older people [37, 65], age is positively associated with COVID-19 prevention behavior (H1b supported). This result confirms past findings of a positive association between age and COVID-19 prevention behavior [44, 66–68] and contradicts findings of a negative [69] or non-significant association [19, 20, 70]. Since COVID-19 is more severe for men than women [37], we also predict that men engage in more COVID-19 disease prevention behavior. However, we find the opposite tendency (H1c not supported), which confirms the results of most studies [19, 20, 44, 48, 66, 68, 69, 71, 72]. This result might be explained by the higher risk aversion of women as compared with men [73, 74], by women’s greater interest in health issues [75], by women’s greater sense of responsibility for their own health [76, 77], and by women’s problem-focused orientation in dealing with the COVID-19 pandemic [48], which all may cause women to act more strongly on their susceptibility to the disease and adopt cautionary behaviors. Hence, beliefs about disease severity, susceptibility to the disease [15], and sensitivity to such beliefs may explain differences in disease prevention behavior.

As additional aspects of vulnerability, we examine physical and psychological health. Regarding physical health, some studies find that chronic health problems are positively associated with disease prevention behavior [67, 69], whereas another study cannot confirm such an association [19]. Unlike these studies, we find that chronic health problems have a U-shaped association with disease prevention behavior (H1a supported). We presume that such problems cause a higher severity of COVID-19 [37], which may trigger disease prevention behavior, and a need to seek regular treatment and thus reduce certain disease prevention behaviors, such as social distancing. Hence, physical health problems may result in the perception of both a higher severity of a disease and a higher barrier to adopting disease prevention behavior, which constitutes a trade-off with a non-linear outcome. Regarding psychological health, studies find positive [19, 32, 44], negative [78], or non-significant [69, 79] associations between anxiety and disease prevention behavior. Moreover, they find a negative [79] or non-significant [78] association between depression and disease prevention behavior. Similar to some of these studies, we find that disease prevention behavior is positively associated with worries (i.e., anxiety) (H1d supported), which reflect a higher perceived susceptibility to or severity of the disease, and negatively associated with depression (H1e supported), which constitutes a barrier in adopting disease prevention behavior. These findings are consistent with research showing that worries trigger health-promoting behaviors (e.g., health screening [80, 81]) and that depression causes negative evaluations of messages received from others [82] and thus triggers disobedience [83]. We also find that disease prevention behavior has non-significant associations with income-oriented work (as Everett et al. [66]), the prevailing climate and population density in the region of birth, and education. Other studies find a positive [44, 67, 69, 71], negative [66, 68], or non-significant [84] association between disease prevention behavior and education.

In addition, we examine how attitudes toward (i.e., perceived benefits of) disease prevention behavior are associated with disease prevention behavior. As the focal disease prevention behaviors reflect a citizen’s compliance with both health guidelines and a national lockdown issued by the government, we examine how disease prevention behavior is associated with attitudes toward the lockdown, the enforcement of the lockdown, and social distancing. We also discuss its association with trust in public institutions. These associations are positive, and most are large (H2a-d supported). This result is consistent with the meta-analytic finding of a significant influence of beliefs about the benefits of disease prevention behaviors on adopting such behaviors [22]. Moreover, this result resembles studies finding positive associations between disease prevention behavior and information appraisal [85], trust in information about disease prevention behavior [78, 86], and benefits of COVID-19 prevention behavior on the adoption of such behavior [8, 20, 67, 87].

In sum, our findings of how health beliefs related to a citizen’s vulnerability and attitude toward disease prevention are associated with the adoption of COVID-19 disease prevention behavior are consistent with past findings. Our original research contributions regarding health beliefs are findings of a non-linear association of chronic health problems and a strong association of lockdown-related attitudes with disease prevention behavior.

Second, as the unique perspective of our study, we draw on collective resilience theory [18] to predict that the character of the COVID-19 pandemic as a national disaster [17] may cause citizens with a particular social orientation to adopt disease prevention behaviors to protect fellow citizens. This perspective differentiates our study from the literature about general and COVID-19-specific disease prevention behaviors, which almost exclusively focuses on self-preservative motives. Using the multidimensional view of individualism and collectivism as measures of a citizen’s orientation toward society [52], we find that the horizontal and vertical dimensions of individualism and collectivism are all positively related to disease prevention behavior (H3a/b supported). Horizontal individualism may promote disease prevention behavior by emphasizing reciprocity [88] and thus actions to protect each other’s health. These findings reveal that people who seek to stand out without desiring special treatment are more inclined to follow COVID-19 disease prevention behaviors. Where egalitarian rules are respected, the horizontal perspective of individualism emphasizes independence and equality among members. Thus, autonomy and independent self-categorization aid the desire to accept personal responsibility for adhering to the COVID-19 restraints imposed by governments [13]. Vertical individualism may promote such behavior by emphasizing competition with others [52], such as the competition for good, prosocial actions conveying social status [89]. Citizens with a sense of such individual competitiveness appear to regard prosocial disease prevention behaviors as a visible, status-conferring benchmark of personal responsibility and self-sacrifice. Horizontal collectivism may promote such behavior through communal sharing of responsibility [88], such as the responsibility to protect society. This finding suggests that horizontal collectivists work together with their in-group and emphasize equal accountability for all group members during the COVID-19 pandemic [13]. Such a cultural inclination might lead to a clear perception that individuals form a strong sense of shared social identity due to their horizontal collectivistic orientation, which enhances their in-group commitment to implement COVID-19 disease prevention behaviors. Vertical collectivism may promote such behavior by emphasizing the duty to support family members [40] and thus to protect their health. Specifically, higher vertical collectivism means a more heightened sense of belonging to the group, social criticism for those who do not conform, and more respect for authority [13]. Consequently, individuals from vertically collectivistic societies encourage their members to engage in the COVID-19 disease preventive behaviors recommended by the government as their representative authority.

Moreover, when focusing on disease prevention behaviors that exclusively benefit other citizens (i.e., covering one’s nose/mouth when sneezing/coughing), but not citizens themselves, the association between the citizen’s social orientation (in particular, collectivism) and such selfless disease prevention behavior becomes dominant. In contrast, the citizen’s own vulnerability is not associated with such selfless disease prevention behavior because it is unrelated to any benefits for other citizens. In addition, the citizen’s attitudes toward the government-imposed lockdown (H2b/c) influence such selfless disease prevention behaviors because these attitudes reflect governmental actions to protect the individual citizens themselves and society as a whole, including fellow citizens. A citizen’s agreement with such governmental actions to protect society may thus also reinforce the citizen’s willingness to participate in these actions and therefore adopt disease prevention behaviors that protect fellow citizens.

Our emphasis on a citizen’s social orientation as a predictor of selfless disease prevention behaviors to protect fellow citizens extends other limited work in this area. Pfattheicher et al. [90] find that empathy positively influences COVID-19 disease prevention behaviors. Moreover, a few studies examine whether personal values influence such behaviors, but their results are mixed. A citizen’s social responsibility [70] and religiosity (Everett et al. [66], not peer-reviewed) positively affect such behaviors, whereas moral standards [79] and the importance of protecting others [70] do not affect such behaviors. These findings resemble our results on the positive association between collectivism and disease prevention behaviors and, taken together, provide evidence for a role of certain aspects of a citizen’s social orientation in the adoption of disease prevention behaviors that benefit fellow citizens. In addition, past studies report that political conservativism negatively affects such behaviors [66], whereas political orientation does not [79]. A separate stream of research examines how not the citizen’s orientation toward society, but conversely the societal pressure on the citizen, affects COVID-19 prevention behaviors. It finds that social pressure has a positive [32, 87] or non-significant association [70].

In sum, we suggest that national pandemics can trigger collective resilience, which causes disease prevention behaviors to be motivated by both self-protection and the protection of fellow citizens. Hence, including social motives and beliefs regarding collective (e.g., governmental) actions can enhance the predictive validity of theories of disease prevention behavior (e.g., the health belief model) that hitherto focused merely on self-preservative motives.

### Practical implications

Our research demonstrates the associations of a citizen’s vulnerability, attitudes toward disease prevention, and social orientation with adopting COVID-19 prevention behaviors.

Regarding vulnerability, we show that female gender, age, and worries are positively associated with such behaviors, whereas depression has a negative association with such behaviors and chronic health problems show a U-shaped association. We recommend increasing the adoption of disease prevention behaviors among male, younger-aged, and unworried citizens by disseminating frequent and meaningful messages that make them more aware of their vulnerability. In particular, the recent literature suggests that gender should be considered in risk communication campaigns to improve the efficacy of messages and the adoption of COVID-19 preventive behaviors [48]. Also, past research shows that regular and meaningful text messages are effective at promoting the adoption of healthy behaviors [8, 91, 92]. Moreover, messages that visualize a person’s vulnerability are effective because they strengthen the perceptions of disease severity and susceptibility to the disease [84, 93, 94]. Similarly, websites, (e.g., online) seminars, MOOC courses, or billboards may be effective ways to disseminate information. In particular, based on Abbas et al. [95], Azadi [96], Maqsood et al. [7], Shoib et al. [86], and Shuja et al. [65], we recommend the development of nationwide health education programs, both offline and online, to promote COVID-19 prevention behaviors. People who are educated about the concepts of the COVID-19 public health crisis are more likely to engage in preventive behaviors, which limits the spread of the disease [97].

Moreover, we recommend increasing the adoption of COVID-19 prevention behaviors among depressed people by addressing their depression. According to past research, effective ways are offering (e.g., online) psychological support and promoting distracting activities (e.g., exercise, communication with colleagues and friends) [98]. Also, as Abbas [46] suggests, since the COVID-19 pandemic has propagated depression on a social level, effective mental and physical public health policies are required, particularly in developing countries, such as Bolivia. These countries have few resources, and their healthcare systems are not sufficiently developed to diagnose sickness promptly. Moreover, in developing countries, poverty in the COVID-19 pandemic magnifies the problem since low socioeconomic status, which is socially frowned upon, is associated with increased rates of suicide [86]. As a result, in comparison to affluent countries, public officials in developing countries face substantial difficulties in dealing with economic poverty, which obstructs the implementation of mental and physical health policies to address the consequences of the COVID-19 pandemic. Following the recommendations of Abbas [46] and Yoosefi et al. [99], we propose the development of public and freely available online and telephonic psychological helplines to make it simple for the general public to obtain mental health counsel from psychologists and other health professionals and to ensure that they can readily communicate to receive the help they require. Moreover, these telephone helplines can also be used for screening and triage services to reduce unnecessary referrals to hospitals during the COVID-19 pandemic [100].

Regarding attitudes toward disease prevention, we find that attitudes toward social distancing, a lockdown, and the enforcement of this lockdown and trust in public institutions have positive associations with COVID-19 prevention behaviors. We recommend frequently reminding citizens about the benefits of disease prevention through advertisements, text messages, or billboards in crowded places (e.g., schools, universities, supermarkets). Moreover, we recommend seeking ways to convince citizens of health guidelines and measures by preparing clear and transparent communication plans, by providing evidence of managerial competence and preparedness, by using trusted spokespeople (e.g., trusted experts), by seeking endorsements for policies from scientists and opinion leaders, and by compensating citizens for adverse effects of these measures (e.g., reduced salary in a lockdown) [101–103]. Threats of punishment for non-compliance with health guidelines and measures are effective [104], but they may also diminish goodwill and the willingness to comply and thus have ambivalent associations [101].

Regarding social orientation, we recommend that policymakers and managers frame the adoption of disease prevention behaviors as contributions to support society or important social groups (e.g., firms, sports clubs, or church communities) because citizens tend to be more motivated to take on behaviors when these have a precise prosocial dimension [105]. According to Kappes et al. [106], leaders should emphasize how lack of compliance with health guidelines adversely affects other citizens’ health. To amplify the mechanisms behind the positive associations of horizontal individualism (reciprocity), vertical individualism (competition), horizontal collectivism (communal sharing), and vertical collectivism (duty to protect family) with disease prevention behavior, we recommend that public officials raise awareness of the benefits of disease prevention behavior for protecting citizens themselves through reciprocal actions, for protecting their families, and for the sharing of responsibilities in society. In the same line, past research suggests framing health guidelines as a moral duty to protect community and family members [13, 105], inducing empathy with other members of society [90, 107], and sending customized messages to social networks of groups [108]. Moreover, we recommend emphasizing that selfless, prosocial disease prevention behavior may increase one’s social status [89], triggering competition for consistent disease prevention behavior. For example, civic responsibility and heightened levels of concern for the health of others, taking precedence over personal freedom and convenience as expected by collectivist norms, are considered reasons for East Asia’s effective control of the COVID-19 pandemic [13, 54]. The above discussion suggests that cultural frames influence behavioral responses to the COVID-19 pandemic at country and individual levels. Therefore, COVID-19 prevention efforts depend not only on how much money a government has or how strict its rules are, but also on public support, cooperation, and cultural orientation.

### Limitations and directions for future research

The strengths of our study are the combination of two theoretical models, a large and nationally representative sample, a reasonably high response rate, and a statistical methodology that disentangles individual and group effects (HLM). Our theoretical approach is original since there are currently few studies using two theoretical models to explain individual and group mental processes to adopt COVID-19 preventive behaviors. As shown in Table 1, most studies restrict their analysis to an individual’s self-protection and disregard people’s social orientation. Moreover, to the best of our knowledge, no such study has ever been conducted in the Americas. Consequently, this research can help close the knowledge gap between developed and developing countries regarding disease prevention behaviors during the COVID-19 pandemic. As another strength, since the data were collected during the first lockdown of Bolivia, individuals experienced for the first time a quarantine lockdown and reflected on their actual recent experiences instead of describing hypothetical situations or recollecting memories.

However, our study has several limitations, such as the focus on only one particular disease (COVID-19) in one country at a specific time. While this makes it difficult to generalize the results across all illnesses in all countries, our research setting constitutes an ideal case for studying the role of collective resilience in a national pandemic, which may cause disease prevention behaviors to be influenced not only by self-preservative motives but also by motives to protect fellow citizens. Future research may seek to analyze similar phenomena in pandemics involving different viruses (e.g., Zika or Ebola), different countries, or other (e.g., more mature) pandemic stages. Moreover, future research may seek to explore various aspects of social orientation and different types of beliefs about collective actions (e.g., concerning the press, vaccination efforts, other public restrictions, or selective exemptions from these restrictions) in the face of a common threat faced by the society.

Another limitation of our study comes from its cross-sectional nature, which makes it difficult to establish a temporal sequence and thus to ascertain causality. Future studies are advised to investigate our hypothesized relationships through longitudinal (e.g., panel) data. For example, Breakwell et al. [10] created the COVID-19 Preventive Behaviors Index (CPBI) to track preventive behaviors over time for policymakers and as a modeling tool for researchers.

As another limitation, we used self-report questionnaires, which makes it difficult to rule out the possibility of respondents’ inherent self-selection bias. Moreover, self-reports are not always honest, and respondents might wish to portray an image of themselves as adhering to current norms and standards because they (erroneously) fear that their responses will expose them and lead to adverse social or even legal consequences. However, this bias might be limited because research by Larsen et al. [109] find no evidence that individuals under-report non-compliant behavior to COVID-19 norms and standards. To account for these possible, limitations, we recommend that future research use different scales, control groups, and clear and concise informed consents.

In addition, future research may develop an integrated spatial-epidemiological approach to develop a geodatabase of COVID-19 preventive behaviors and the associated factors, including the determinants proposed in this research (e.g., Mohammadi et al. [110]). This geodatabase will allow public health officials to develop appropriate and timely interventions in areas with high priority.

## Conclusions

The study makes two main contributions to the literature. First, because the COVID-19 pandemic is a national tragedy, citizens with a particular social orientation are more likely to engage in disease prevention behaviors. This finding is an original contribution of our research based on collective resilience theory. Second, based on the health belief model, we identified a non-linear association between chronic health problems and disease prevention behaviors. Moreover, we also discovered a strong connection between views toward a national lockdown and disease prevention behaviors. These findings suggest that an individual’s social orientation influences behavior in response to the COVID-19 pandemic at the social and individual levels. Consequently, COVID-19 preventive public efforts are dependent not only on a government’s financial resources or the strictness of its legislation, but also on public support, cooperation, and social norms.

Finally, when a transmissible viral infection recurs, it dramatically reduces worldwide mobility and business. Even 2 years after the beginning of the multiple waves of COVID-19 infection, public health and economic crises are still persistent worldwide [111]. Therefore, following the relaxing of lockdowns worldwide and intensive vaccination programs, there is a need for coordinated public health strategies to return to normality but without disregarding disease prevention behaviors. Our conceptual framework and results suggest that a proper management of public health crises requires public officials to develop strategies that consider the complexity of human behavior through a combination of individual and socially oriented policies. Moreover, our research aims to inspire academics to interpret and use the COVID-19 pandemic as a transformative tool for innovation by reshaping and redesigning their research methodologies based on a new normal. The COVID-19 pandemic will subside when society as a whole and every citizen as an individual follow recommended prevention behaviors. As Albert Camus wrote in his book ‘The Plague’ [112]: *“This whole thing is not about heroism. It’s about decency. It may seem a ridiculous idea, but the only way to fight the plague is with decency … that it consists in doing my job.”*

## Supplementary Information

**Additional file 1.**

## Data Availability

The datasets used and/or analyzed during the current study are available from the corresponding author on reasonable request.
