# Supplementary material for: Explaining interpersonal differences in COVID-19 disease prevention behavior based on the health belief model and collective resilience theory: a cross-sectional study from Bolivia
Source: BMC Public Health. 2022 May 31;22:1077. doi: 10.1186/s12889-022-13068-1 (PMC9153240; doi:10.1186/s12889-022-13068-1)
Supplement: Supplementary file 1 — Additional file 1. [file 12889_2022_13068_MOESM1_ESM.docx]

**Supplementary material. Construct scales and their literature sources.**
